# The mutation spectrum in familial versus sporadic congenital cataract based on next-generation sequencing

**DOI:** 10.1186/s12886-020-01567-x

**Published:** 2020-09-03

**Authors:** Fan Fan, Yi Luo, Jihong Wu, Chao Gao, Xin Liu, Hengjun Mei, Xiyue Zhou

**Affiliations:** 1 Department of Ophthalmology, Eye and Ear, Nose, and Throat Hospital, Fudan University, Shanghai, China; 2 Eye Institute, Eye and Ear, Nose, and Throat Hospital, Fudan University, Shanghai, China; 3 Key Laboratory of Myopia, Chinese Academy of Medical Sciences, Shanghai, China

**Keywords:** Congenital cataract, Gene mutation, Sporadic, Familial, NGS, Mutation spectrum, Next-generation sequencing

## Abstract

**Background:**

Congenital cataract (CC) is a significant cause of lifelong visual loss, and its genetic diagnosis is challenging due to marked genetic heterogeneity. The purpose of this article is to report the genetic findings in sporadic and familial CC patients.

**Methods:**

Patients (*n* = 53) who were clinically diagnosed with CC and their parents were recruited. Blood samples were collected in our hospital. Mutations were detected by panel-based next-generation DNA sequencing (NGS) targeting 792 genes frequently involved in common inherited eye diseases.

**Results:**

We identified variants in 10/37 cases (27.02%) of sporadic CC and 14/16 cases (87.5%) of familial CC, which indicated a significant difference (*P* = 0.000). Of the 13 variants identified in sporadic cases, nine were previously reported mutations, and three were novel mutations, including one de novo mutation (*CRYBB2* c.487C > T). The most frequent variants in our cohort were in crystallins and cytoskeletal genes (5/27, 18.52%), followed by proteins associated with X-linked syndromic conditions (14.81%) and transcriptional factors (11.11%). Additional information on the possibility of complications with inherited ocular or systemic diseases other than CC was provided in 17/27 (62.96%) variants.

**Conclusions:**

These results contribute to expanding the mutation spectrum and frequency of genes responsible for CC. Targeted NGS in CC provided significant diagnostic information and enabled more accurate genetic counselling. This study reports the different distributions of mutation genes in familial and sporadic CC cases.

## Background

Congenital cataracts (CCs) are now the most common avoidable cause of childhood blindness worldwide, accounting for 10–35% of such cases, with an estimated incidence of 0.63–9.14/10,000 births [1–4]. Management is often difficult due to the risk of amblyopia in the developing visual system and complications of glaucoma, posterior synechia or visual axis opacification, which require additional surgery [5]. CCs occur due to the disruption of the lens microarchitecture or the protein function in the lens [6]. Except for a very few infectious cases, only one-third of CC cases have a positive family history [7], with the other two-thirds having an unknown aetiology [8]. Therefore, a significant proportion are sporadic cases in which it is not known whether there is an underlying genetic cause for the lens abnormality.

Thus far, approximately 350 genes have been reported to be associated with CC (Cat-Map; http://cat-map.wustl.edu/); these include mutations in crystallins and gap junction, membrane transport and channel, and cytoskeletal proteins and growth and transcription factors [9]. Locating and identifying the involved genes and mutations are essential to gaining an understanding of the molecular defects and pathophysiologic characteristics underlying inherited CC.

A conventional approach to identifying mutations in CC is usually performed by Sanger sequencing only in familial cases and is time-consuming and costly, with a detection rate of 30–50% in apparent autosomal dominant cases [10, 11]. Due to marked genetic and phenotypic heterogeneity, determining the precise genetic cause of CC and establishing a robust genotype-phenotype correlation is challenging. Next-generation DNA sequencing (NGS) is increasingly powerful as a diagnostic tool and offers speed, precision, and cost-effectiveness for heterogeneous conditions [12]. This has been demonstrated in studies to determine the cause of other heterogeneous inherited eye diseases, such as congenital macular dystrophy and retinal pigmentosa [13–16]. Recent studies have also shown that NGS allows the efficient identification of genetic causes of CC in the majority of cases, thereby improving its diagnosis and clarifying inheritance patterns [17–19] while guiding genetic counselling and increasing prognostic accuracy.

In this study, we applied targeted NGS in 792 genes involved in common inherited eye diseases to detect causal mutations in a relatively large series of CCs, including a high proportion of sporadic cases, and report the different distributions of mutated genes in sporadic versus familial CC cases (sCC VS fCC), while broadening the mutation spectrum and frequency of genes responsible for CC.

## Methods

### Ethical statement

All participants (parents on behalf of their children) provided written informed consent forms for both genetic counselling and molecular genetic testing prior to enrolment. The study was approved by the Ethics Committee of the Eye and ENT Hospital of Fudan University. All research was conducted in accordance with the Declaration of Helsinki.

### Clinical evaluations and sample collection

Patients who were clinically diagnosed with CC from June 2018 to May 2019 were recruited. All patients underwent a detailed ophthalmic examination, including slit-lamp examination, B ultrasound, intraocular pressure measurement, and ultrasonic A-scan, as mentioned in our previous study [20]. Visual acuity (VA) was recorded in all patients who were able to cooperate. Patients diagnosed with monocular CC additionally underwent post-eyeball colour Doppler ultrasound to help in the differential diagnosis of persistent hyperplastic primary vitreous (PHPV). Children younger than 3 years old were examined under sedation with chloral hydrate. The lens phenotypes of patients and their parents were carefully recorded in all families and included childbirth history, medical history, family history and a detailed history of the gestation period, including high fever, rubella virus [RV] TORCHES ([*Toxoplasma gondii*; *T*. *gondii*], cytomegalovirus [CMV], herpes simplex virus [HSV], syphilis [caused by *Treponema pallidum*]), tuberculosis infection, exposure to radiation, and drug intake. Additional systemic problems were also recorded in patients and included serum biochemical tests for levels of blood glucose, calcium and phosphorous as well as urine tests. The probands for whom at least one immediate family member had a history of CC were defined as familial cases. Those who had no family history and had been excluded from infection factors were classified as sporadic cases. Blood samples were collected in children while under general anaesthesia during eye surgery and from their biological parents (Trio sequencing) in our hospital. In familial cases, blood samples of other available affected relatives were also collected.

### Next-generation sequencing

Genomic DNA was extracted from peripheral blood samples using standard methods. Panel-based NGS was performed on all subjects in this study. We designed the Target_Eye_792_V2 chip with exon-capture and untranslated regions (UTRs) of 792 genes most frequently involved in common inherited eye diseases (Additional file 1, Table S1), which were produced by BGI-Shenzhen, Guangdong, China as previously reported [21]. Then, DNA fragments were sequenced by an Illumina HiSeq 2000 platform (Illumina, Inc., San Diego, CA, United States). The following databases were then used to annotate all identified variants with a minor allele frequency (MAF) > 0.1% to eliminate benign variants as previously described [22]: dbSNP1371 (http://hgdownload.cse.ucsc.edu/goldenPath/hg19/database/snp137.txt.gz), HapMap Project (ftp://ftp.ncbi.nlm.nih.gov/hapmap), 1000 Genomes Project (ftp://ftp.1000genomes.ebi.ac.uk/vol1/ftp), YH database (http://yh.genomics.org.cn/), and Exome Variant Server (http://evs.gs.washington.edu/EVS/). Subsequently, variant prioritization was performed to combine the total depth, quality score, MAF, potential deleterious effect and existence of mutation reports in common databases such as the Human Gene Mutation Database (HGMD), ClinVar or Online Mendelian Inheritance in Man (OMIM). Finally, variants were classified as pathogenic, likely pathogenic, uncertain significance according to the American College of Medical Genetics (ACMG) and genomics guidelines. Sanger sequencing was performed to confirm the candidate variants.

## Results

### Participant characteristics

A total of 152 subjects of 53 families were recruited in this study, including 16 familial cases (49 subjects in total) and 37 sporadic cases (106 subjects in total). Parental samples in one familial case and six sporadic cases were not completely obtained for some reasons beyond control. All the familial cases had at least one affected parent (11 mothers and 5 fathers). In addition, the available affected immediate relatives, the brother, the paternal grandfather and the maternal grandmother in three familial cases participated in the test. The mean ages of the 53 children and their mothers and fathers were 3.0 [1.50–6.00], 30.72 ± 5.02, and 32.65 ± 5.19 years old, respectively. There were more binocular cases than monocular cases and more male than female cases. More detailed information is presented in Table 1. No significant differences were found between sCC and fCC in the mean ages of children and the parents or other constituent ratios (*P* values are presented in Table 1).

**Table 1:** Basic characteristics of the participants in our study

	sCC	fCC	*P* value
Number of patients	37	16	
Male:female	21:16	10:6	0.623
Mean age of patients	3.00 (1.50–6.00)	3.00 (1.63–6.75)	0.133
mothers	31.26 ± 4.97	29.00 ± 4.88	0.434
fathers	33.75 ± 5.35	30.71 ± 4.38	0.324
Binocular:monocular	26:11	15:1	0.067
Detected cases	10/37 (27.03)	14/16 (87.50)	**0.000**
Detected variants	11	16	

Values are shown as n (%) and medians (IQRs) or medians± standard deviation for normally distributed data

Bold text is used for *p* values under 0.01, indicating statistical significance

### Variants identified

A total of 27 variants were found in 24 of the 53 patients with CC in our cohort, yielding a total detection rate of 45.30%. We identified variants in 10/37 (27.03%) sCC and 14/16 (87.5%) fCC cases, indicating a significant difference (*P* = 0.000, Table 1). The detection rate was lower in monocular cases (4/12, 33.33%) than in binocular cases (20/41,48.80%), but the difference was not significant (*P* = 0.512).

The variants detected are presented in Table 2 and Table 3. According to the ACMG mutation guidelines, 17 of 27 variants were classified as pathogenetic, five were likely pathogenic, and seven were uncertain significance (VUS).

**Table 2:** Detected variants in sporadic cases

Family	Phenotype	Inheritance:Before/After testing	Gene	Refseq ID	Nucleotide change	Predicted amino acid change	Heterozygosity	Segregation	Pathogenicity	In silico Prediction				Note (other reported phenotype or references)
										FATHMM	SIFT	Mutation Taster	LRT	
1	Bi, Sub, Junvenile	Sporadic/AR	*CYP27A1*	NM_000784.3	c.1263 + 1G > A	–	Hom	Father heterozygous	P	/	/	D	/	Cerebrotendinous xanthomatosis [23]
2	Bi,All	Sporadic/new AD	*CRYBB2*	NM_000496.2	c.487C > T	p.Gln163*|p.Q163*	Het	De novo; not found in parents	P	/	/	D	D	Novel
3	Bi,All	Sporadic/AD?	*OPA3*	NM_001017989.2	c.123C > G	p.Ile41Met|p.I41M	Het	Present in unaffected mother Present in unaffected mother	P	D	D	D	T	Optic atrophy [24]
			*JAG1*	NM_000214.2	c.1511A > G	p.Asn504Ser|p.N504S	Het		P	B	B	D	D	Alagille syndrome [25, 26]
4	Mo,Sub + Post,	Sporadic/AD or AR?	*BEST1|BEST1*	NM_001139443.1|NM_004183.3	c.20G > A	p.Ser7Asn|p.S7N	Het	Present in unaffected mother	P	D	B	D	T	Best vitelliform macular dystrophy [27, 28]
5	Mo, All, PHPV	Sporadic/AD or AR?	*BEST1*	NM_001139443.1|NM_004183.3	c.584C > T	p.Ala195Val|p.A195V	Het	Present in unaffected father	P	D	D	D	D	Bestrophinopathy [29, 30]
6	Bi,Nuc	Sporadic/X-linked?	*NHS*	NM_001291867.1	c.2774_2775dup	p.Gln926Leufs*3	Hemi/Het	Present in unaffected mother	LP	/	/	/	/	Novel
7	Bi,OD-Dot,OS-Ant+Dot	Sporadic/X-linked?	*NHS*	NM_001291867.1	c.2933 T > C	p.Ile978Thr|p.I978T	Het/Hemi	Present in unaffected father	VUS	B	B	D	D	Novel
8	Mo,Sub, Junvenile	Sporadic/AR?	*WFS1*	NM_006005.3	c.2603G > A	p.Arg868His	Het	Present in unaffected father	LP	D	D	D	D	Wolfram-like syndrome [31–33]
9	Mo,Nuc	Sporadic/XR	*COL4A5*	NM_033380.2	c.4003C > T	p.Pro1335Ser|p.P1335S	Hemi/Het	Yes/Present in son and unaffected mother	VUS	D	D	D	D	Alport Syndrome [34]
10	Bi,Cort+Sub,FEVR, Cleft Lip and Palate	Sporadic/AD?	*TSPAN12*	NM_012338.3	c.194C > T	p.Pro65Leu|p.P65L	Het	Present in unaffected father	VUS	B	B	D	D	Novel, gene associated with FEVR [35]

*Bi* binocular, *Mo* monocular, *Sub* subcapsular, *Cort* cortical, *Post* posterior polar, *Nuc* nuclear, *Ant* anterior polar, *All* all white, *Dot* dot-like, *Peri* perinuclear, *Micro* microphthalmia, *Hom* homozygosis, *Het* heterozygosis, *P* pathogenic, *LP* likely pathogenic, *VUS* variant of unknown significance, *D* damaging, *B* benign, *T* tolerated

**Table 3:** Detected variants in familial cases

Family ID	Phenotype	Inheritance Before/After testing	Gene	Refseq ID	Nucleotide change	Predicted amino acid change	Heterozygosity	Segregation	Pathogenicity	In silico prediction				Note
										FATHMM	SIFT	Mutation Taster	LRT	
1	Bi,All	AD/AD	*CRYGC*	NM_020989.3	c.497C > T	p.Ser166Phe|p.S166F	Het	Yes/present in affected mother	P	D	D	D	D	[19]
2	Bi,Lam	AD/AD	*CRYGD*	NM_006891.3	c.70C > A	p.Pro24Thr|p.P24T	Het	Yes/present in affected father	P	B	B	/	/	[36, 37]
3	Bi, OS-All; OD-Dot	AD/X-linked	*BCOR*	NM_001123385.1	c.3490C > T	p.Arg1164*|p.R1164*	Het	Yes/present in affected mother	P	/	/	D	/	OFCD [38]
4	Bi, Nu	AD/AD	*CRYAA*	NM_000394.3	c.61C > T	p.Arg21Trp|p.R21W	Het	Yes/present in affected mother	P	D	D	D	D	[37, 39]
5	Bi,Cora	AD/AD	*CRYGD*	NM_006891.3	c.70C > A	p.Pro24Thr|p.P24T	Het	Yes/present in affected mother	P	B	B	/	/	[36, 37]
6	Bi, Nu	AD/AD	*BMP4*	NM_001202.4	c.751C > T	p.His251Tyr|p.H251Y	Het	NK/present in unaffected mother	P	B	D	D	D	Microphthalmia [40]
			*CRYBA1*	NM_005208.4	c.626C > G	p.Ser209Trp|p.S209W	Het	Yes/present in affected father and paternal grandfather	P	D	D	D	D	[17]
7	Bi,All	AD/AD	*CRYGC*	NM_020989.3	c.192del	p.Asp65Thrfs38|p.D65Tfs38	Het	Yes/present in affected mother and elder brother	P	/	/	/	/	Adjacent loci c.193del [41]
8	Bi,Peri+Cor	AD/?AD or AR	*OPA3*	NM_001017989.2	c.123C > G	p.Ile41Met|p.I41M	Het	Yes/present in affected mother	LP	D	D	D	T	Optic atrophy [24]
9	Bi+Micro	AD/AD	*GJA8*	NM_005267.4	c.136G > A	p.Gly46Arg|p.G46R	Het	Yes, present in affected father	LP	D	D	D	D	[42]
10	Bi+Dot	AD/AD	*PAX6*	NM_001310158.1	c.52G > A	p.Gly18Arg	Het	Yes, present in affected mother	LP	D	D	D	T	Peter’s [43]
11	Bi All	AD/AD	*PAX6*	NM_001310158.1	c.113G > C	p.Arg38Pro|p.R38P	Het	Yes/presented in affected mother from a consanguineous family	VUS	D	D	D	T	c.113G > A (p.R38Q) CC and nystagmus (HGMD)
12	Bi,Nys,Post +OD-ASD	AD/AD	*PAX6*	NM_001310158.1	c.966del	p.Phe323Serfs56|p.F323Sfs56	Het	NK/parental samples unavailable, mother affected	P	/	/	/	/	Aniridia [44]
13	Bi,Post+Sub	AD	*CYP1B1*	NM_000104.3	c.319C > G	p.Leu107Val|p.L107V	Hom/het	Present in affected father and unaffected mother	VUS	/	/	D	D	Glaucoma [45]
			*FBN1*	NM_000138.4	c.7559C > T	p.Thr2520Met|p.T2520M	Het	Present in affected father	VUS	D	B	D	D	Marfan [46]
14	Bi	AD/AD	*WFS1*	NM_006005.3	c.449C > T	p.Ala150Val|p.A150V	Het	Present in affected mother	VUS	D	B	D	D	Wolfman-like syndrome [31–33]

*Bi* binocular, *All* all white, *Lam* lamellar, *Sub* subcapsular, *Cort* cortical, *Post* posterior polar, *Nuc* nuclear, *ASD* anterior segment dysplasia, *Dot* dot-like, *Peri* perinuclear, *Micro* microphthalmia, *Cora* coralliform, *Nys* nystagmus, *Hom* homozygosis, *Het* heterozygosis, *P* pathogenic, *LP* likely pathogenic, *VUS* variant of unknown significance, *D* damaging, *B* benign, *T* tolerated, *NK* not known, *OFCD* Oculofaciocardiodental syndrome

We identified three novel likely cataractous causative mutations in sCC in *CRYBB2* and NHS (*2), one of which was a de novo mutation in *CRYBB2* c.487C > T (p. Gln163*|p. Q163*). Eight of the 27 variants detected in our cohort were previously reported pathogenic gene mutations in CC, including loci in *CRYGC*, CRYGD(*2), *CRYAA*, *CRYBA1*, and *GJA8* and adjacent loci in *CRYGC* and *PAX6*. Another 16 variants involved in additional ocular or systemic diseases that had been reported or included by HGMD or ClinVar were also identified, including *CYP27A1*, *OPA3*, *JAG1*, *BEST1*, *BMP4, CYP1B1*, and *TSPAN12* (see Tables 2 and 3, Note column).

In terms of gene function, genes encoding crystallins were the most frequently identified in our cohort, accounting for 7/27 (25.93%) of the cases, followed by cytoskeletal proteins (18.52%), X-linked syndromic proteins (14.81%) and transcriptional factors (11.11%).

### Differential distribution of mutational genes

**Fig. 1:**
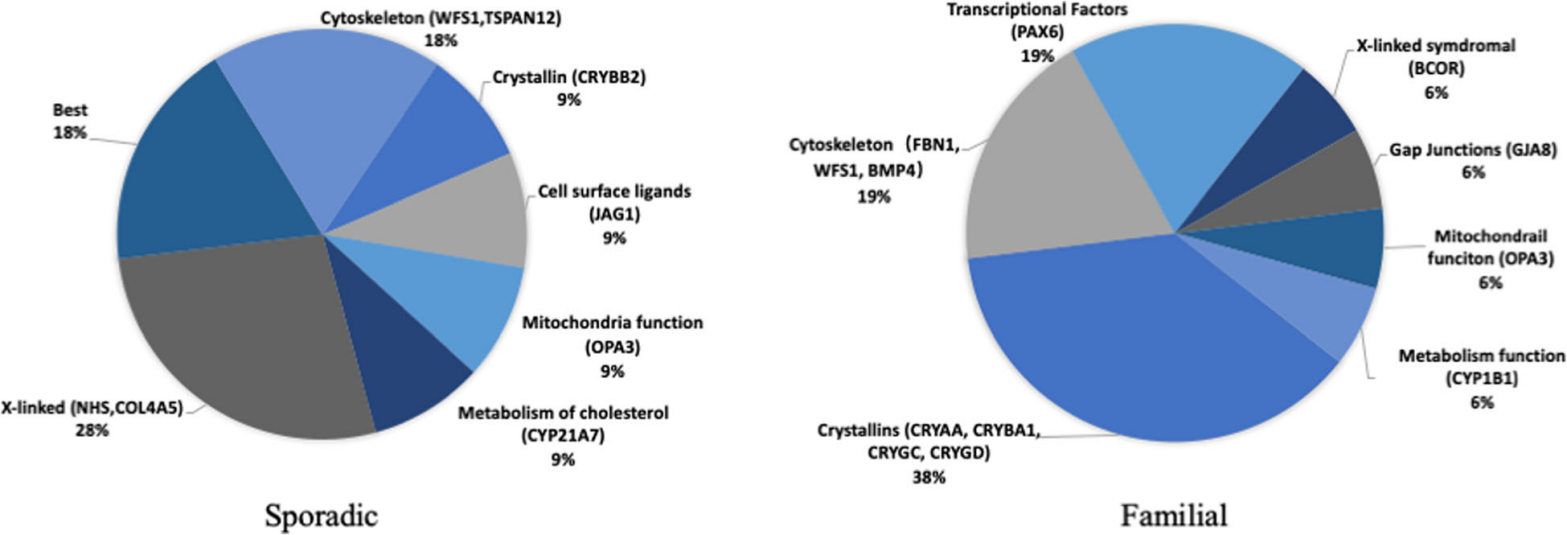
Different distributions of mutational genes in familial versus sporadic congenital cataracts

A comparison of the distributions of mutational genes between fCC and sCC showed that variants in crystallins accounted for the highest proportion (37.50%) in fCC cases but only 9.00% in sCC cases (Fig. 1). The sporadic cases mainly consisted of X-linked syndromic proteins and structural protein genes, including transmembrane and collagen-associated proteins.

## Discussion

Approximately 70% of CC cases may occur alone, and 15% of such cases may be accompanied by other ocular abnormalities, such as microphthalmia, aniridia, or retinal degeneration. In another 15% of cases, cataracts are one part of a multisystem genetic disorder [47]. To obtain clues related to the noncataractous phenotype, we designed a panel with exon-capture and NGS targeting of the 792 genes most frequently involved in common inherited eye diseases. Compared to related previous studies, our study included the largest numbers of patients and targeted genes. We achieved detection rates in familial and sporadic cases similar to those in a recent study [37]. Although the overall detection rate (45.3%) in our cohort was apparently lower than that in the other studies listed in Table 4, these rates are not comparable due to differences in the proportions of participants. Most of the studies [17–19] included only binocular cataracts, whereas we enrolled many monocular cases. Regarding the distribution of genes, our result was slightly different from those reported previously. Li et al. [37] reported that variants in the crystallin genes were the most frequent mutations found in their study, whether in familial or sporadic cases. We found that variants in crystallins accounted for a similar proportion of fCC cases but only 1 sCC case (Fig. 1). X-linked syndromic proteins and structural protein genes, such as transmembrane and collagen-associated proteins, accounted for most of the sCCs in our study.

**Table. 4:** Studies related to the mutation spectrum of CC obtained using NGS in the past 5 years

	Our cohort	Li et al., 2018 [37]	Gillespie et al., 2014 [17]	Ma et al., 2016 [19]	Zhai et al., 2017 [18]
Target genes	792 inherited eye diseases	80 cataract-associated genes	115 genes associated with CC	32 cataract -associated genes	54 cataract-associated genes
Detection rate	Familial, 87.5% sporadic, 27.03%	familial, 75% sporadic, 47.8%	70%	70%	62.96%
Participants	38 sporadic and 16 familial cases, 42 bilateral and 12 unilateral	23 sporadic and 16 familial cases, all bilateral	15 sporadic and 21 familial cases all bilateral 16 syndromic	24 sporadic and 22 familial cases all bilateral nonsyndromic	25 familial and 2 sporadic

In our study, approximately 17/27 (62.96%) variants provided clues regarding the possibility of complication with inherited ocular or systemic diseases other than CC. Among these, nine identified loci provided additional ophthalmological diagnostic information. For instance, *OPA3* mutations are associated with optic atrophy [24], *BEST1* mutations are associated with best vitelliform macular dystrophy (BEST) [27–30], *TSPAN12* mutations are associated with familial exudative vitreoretinopathy (FEVR) [35], *PAX6* mutation are associated with aniridia and Peter’s anomaly [48], and *CYP1B1* mutations are associated with glaucoma [45]. In addition, we also identified a monoallelic mutation in *BMP4*, which has been mostly associated with microphthalmia [40] or facial clefts [49]. Eight variants were associated with systemic syndrome. *WFS1* is the most common causative gene in Wolfram-like syndrome, a rare autosomal dominant disease characterized by congenital progressive hearing loss, diabetes mellitus, and optic atrophy [50]. *COL4A5* is one of the causative genes in Alport Syndrome, a genetic condition characterized by progressive loss of kidney function, hearing, and eye abnormalities, including misshapen lenses and abnormal retina [34]. *JAG1* has been associated with Alagille syndrome, which involves cholestasis, cardiac defects, ocular abnormalities, skeletal abnormalities and characteristic faces. Loss-of-function mutations in the *BCOR* gene have been identified in individuals with oculo-facio-cardio-dental syndrome (OFCD), which includes microcornea, CC, and facial, cardiac, and dental abnormalities [38]. Mutations in the *FBN1* (fibrillin-1) gene may be diagnostic of Marfan syndrome [46]. *NHS* mutations have been identified in patients with Nance-Horan syndrome (NHS), an X-linked developmental disorder characterized by CC, dental anomalies, facial dysmorphism and, in some cases, mental retardation [51]. Clinically, a new diagnosis was made after surgery and with reference to genetic testing in at least two patients in our cohort. One of the sporadic cases (ID 10 in Table 2) presented some retinal abnormalities during operations after the removal of cataracts in both eyes, including settled subretinal exudates and dragging of the optic disc. Combined with this clinical manifestation, we have clarified the diagnosis of FEVR with regard for the *TSPAN12* mutation, which is a pathogenic gene known to indicate FEVR. We also observed dental, facial and mental anomalies and made a new diagnosis of NHS at 2 years after the first CC operation was performed in one of the sporadic cases with an identified *NHS* mutation (ID 6 in Table 2). However, whether other variants are associated with a noncataractous phenotype is difficult to confirm. For example, in family 3 (Table 2), we cannot clearly ascribe one of these variants, *OPA3* or *JAG1* to a cataractogenic effect. It is possible that one or more gene mutations cause multiple eye abnormalities at the same time, and cataracts are only one of the first manifestations found in the clinic. During the follow-up period after cataract surgery, we will pay more attention to whether the child tends to experience optic atrophy and will give suggestions for monitoring liver and cardiac function. The relationships between complicated phenotypes and mutations in ocular genes are not explicit. Thus, more cases should be included, and more experiments should be performed to verify these connections.

It is worth emphasizing that those identified variants in non-classical cataract genes may not be initially ascribed to a cataractogenic effect. They might indicate other inherited eye disorders or syndromes, in which a cataractous phenotype may not be presented in every carrier. Another possibility was that the exact cataractous causative genes are located in regions that have not yet been detected, or even that the cataractous phenotype was not caused by genetic factors at all or may involve epigenetic factors.

The phenomenon in which identified heterozygous variants are also present in unaffected parents in sporadic cases (Table 2) might be explained by incomplete and variable penetrance; the underlying mechanisms of this phenomenon remain largely unknown. A recent study also provides support showing that variants associated with inherited eye disorders are frequently encountered in unaffected individuals and that one in six genes implicated in inherited eye disorders are potentially associated with variable penetrance [52]. The number of variants and genes that do not segregate (Table 2) is relatively high in our study. Some of these genes, such as *BEST1* and *WFS1*, were shown to exhibit variable penetrance in a previous study [50]. Incomplete penetrance of the remaining genes might not be supported by sufficient evidence, or these genes might not be the causative genes. This phenomenon might also be due to the limited number of samples detected. In a future study, we will continue to expand the sample size, collect more samples of family members, and improve the history tracking. We believe that the proportion of this phenomenon will be significantly reduced.

This study emphasizes the power and necessity for trio NGS analyses of CC families. By identifying pathogenic heterozygous and homozygous mutations, de novo mutations, and parental mosaicism, such analyses may reveal a new pattern of inheritance in CC with significance not limited to the affected child. However, trio NGS can reveal numerous VUS, for which functional validation is mandatory, although it is still a challenge. Furthermore, future research is required to determine the clinical significance of non-Mendelian inheritance, the intricate mutual effect between genetic predispositions and environmental factors, and interactions between genetic and epigenetic. These studies will provide important insights into the pathogenesis and the complex genotype-to-phenotype association of CC. In the future, these results may also lead to the development of novel gene therapies for some types of congenital cataracts, similar to other inherited eye diseases.

A limitation of this study is that samples in which no mutations were identified could be further submitted to whole-genome sequencing but rarely are because it is challenging to obtain a sufficient amount of blood from infants and young children to meet experimental needs.

## Conclusions

In conclusion, our study highlights the benefits of an NGS approach combined with the analysis of a large targeted group of genes in a setting of genetically heterogeneous CC patients. Our findings provide significant diagnostic information and enable more accurate genetic counselling. Our results expand what we know about the mutation spectrum and frequencies of genes responsible for CC as well as the different distributions of genes mutated in familial and sporadic cases in the Chinese population.

## Supplementary information

**Additional file 1:** **Table S1.** Gene list of capture panel. 792 genes most frequently involved in common inherited eye diseases.

## Data Availability

The raw sequencing datasets generated during the current study are not publicly available due to legal requirements in accordance with The Regulations of People’s Republic of China on Administration of Human Genetic Resources. However, original analyzed data of the sequencing datasets (excluding patient information) are available from the corresponding author on reasonable request.

## Abbreviations

CC: Congenital cataract
sCC: Sporadic congenital cataract
fCC: Familial congenital cataract
NGS: Next-generation DNA sequencing
HGMD: Human Gene Mutation Database
ACMG: American College of Medical Genetics
VUS: Uncertain significance
FEVR: Familial exudative vitreoretinopathy
BEST: Best vitelliform macular dystrophy
NHS: Nance-Horan syndrome
